# Comparing the therapeutic efficacy of endoscopic minimally invasive surgery and traditional surgery for early-stage breast cancer: A meta-analysis

**DOI:** 10.1515/med-2024-1133

**Published:** 2025-01-20

**Authors:** Qiyi Ma, Tingting Shi, Huan Wang, Jie Xing

**Affiliations:** Department of Breast and Nail Surgery, The Affiliated Hospital to Changchun University of Chinese Medicine, Changchun 130021, Jilin, China; Treatment Center, The Affiliated Hospital to Changchun University of Chinese Medicine, Changchun 130021, Jilin, China; Rehabilitation Department, The Affiliated Hospital to Changchun University of Chinese Medicine, Changchun 130021, Jilin, China

**Keywords:** early-stage breast cancer, endoscopic minimally invasive surgery, traditional surgery, meta-analysis

## Abstract

**Background:**

Early-stage breast cancer requires effective surgical interventions. This meta-analysis compares the therapeutic efficacy of endoscopic minimally invasive surgery (EMIS) with traditional surgery, such as modified radical mastectomy.

**Methods:**

Following Preferred Reporting Items for Systematic Reviews and Meta-Analyses guidelines and the Patient, Intervention, Comparison, Outcome model, we systematically searched PubMed, Embase, Web of Science, and the Cochrane Library until July 19, 2023. We included comparative trials, focusing on randomized controlled trials, retrospective, and prospective studies. Metrics analyzed included operative time, blood loss, postoperative drainage volume, and lymph node harvest using Stata version 17.

**Results:**

Out of 943 studies, six met the inclusion criteria. Endoscopic surgery had a longer operative time (weighted mean difference [WMD] = 1.03, *P* < 0.01) but significantly less blood loss (WMD = −1.48, *P* < 0.01). No significant differences were noted in drainage volume and lymph node harvest.

**Conclusions:**

EMIS reduces intraoperative blood loss but requires more time than traditional surgeries. Both methods show comparable outcomes in postoperative drainage and lymph node harvest, supporting their efficacy in treating early-stage breast cancer.

## 1 Introduction

Breast cancer is a highly common kind of cancer in women globally, requiring effective and well-tolerated treatments. Surgical resection continues to be a fundamental aspect of the treatment approach, particularly for breast cancer in its early stages [1,2]. Conventional surgical methods, including modified radical mastectomy (MRM), effectively remove tumors but can lead to unsatisfactory cosmetic results, such as prominent and noticeable incisions and changes to the natural contour of the breast. As breast cancer mostly impacts women, there is a growing emphasis on the psychological and aesthetic consequences after surgery [3]. The pursuit of ideal therapeutic results has now started to include not only the requirement for cancer safety, specifically total removal of the tumor and minimal chance of it coming back locally or throughout the body, but also a visually pleasing outcome after the surgery. This combined requirement has resulted in the emergence and increasing prevalence of breast-conserving surgery (BCS) as a substitute for conventional mastectomy treatments. Research has confirmed that BCS provides comparable or even better rates of disease-free survival (DFS) and local recurrence compared to standard surgical procedures. Additionally, BCS preserves the important anatomical structures of the breast, particularly the nipple-areolar complex [4,5].

The use of endoscopic procedures into minimally invasive surgery represents a notable shift in the field, driven by advancements in medical research. Endoscopic procedures, which were first widely used in other surgical fields, are now becoming more frequently used to treat early-stage breast cancer. This procedure provides the dual benefit of efficient cancer therapy, in line with conventional surgical methods, together with a visually superior outcome [6,7]. Endoscopic surgery allows for the removal of tumors and examination of lymph nodes using smaller incisions that are strategically placed in less noticeable areas, such as the axillary region. This approach satisfies patients’ aesthetic concerns while ensuring complete tumor removal and the ability to perform lymph node biopsies or dissections [8].

Despite these developments, there are still uncertainties about the comparative effectiveness of endoscopic minimally invasive surgery (EMIS) compared to standard surgical procedures for treating early-stage breast cancer. The genuine therapeutic index of these new treatments must be systematically evaluated by comparing their potential for complete tumor excision and maintenance of aesthetic aspects post-operation with established approaches. The intricate interaction of these factors necessitates a thorough systematic review and meta-analysis to compare the effectiveness of EMIS with traditional surgical procedures in treating early-stage breast cancer.

## 2 Materials and methods

### 2.1 Search strategy

In the conduct of this meta-analysis and the subsequent dissemination of its findings, strict compliance was observed with the guidelines outlined in the Preferred Reporting Items for Systematic Reviews and Meta-Analyses [9]. The study followed the Patient, Intervention, Comparison, Outcome (PICO) framework to define the following variables: the patient cohort included women diagnosed with early-stage breast cancer; the intervention of interest was EMIS; and the comparison was made against traditional breast cancer surgical techniques, including both MRM and BCS. The outcomes evaluated included therapeutic efficacy measures such as tumor excision completeness, local recurrence rates, DFS, and postoperative aesthetic outcomes. On November 27, 2024, a comprehensive literature search was executed across four digital repositories – PubMed, Embase, Web of Science, and the Cochrane Library – without imposing any temporal restrictions. The search strategy was meticulously crafted around select key terms including, but not limited to, “early-stage breast cancer,” “endoscopic minimally invasive surgery,” and “traditional surgery.” These terms were judiciously chosen to encapsulate the expansive ambit of the PICO framework, thereby ensuring an exhaustive retrieval of germane studies pertinent to this meta-analysis. Additionally, no restrictions concerning language were instituted, and hand-searching was performed on the bibliographies of pertinent articles to identify any auxiliary publications that may be of relevance. The detailed search strategies for each database, including search terms, Boolean operators, and filters used, are provided in Table A1 for further reference.

### 2.2 Inclusion criteria and exclusion criteria

In the execution of this meta-analysis, explicit inclusion and exclusion criteria were employed to facilitate the selection of relevant studies. The criteria were formulated as follows.

Inclusion criteria included: (1) intervention: studies that conducted comparative trials on the clinical efficacy of EMIS versus traditional BCS for treating early-stage breast cancer; (2) study population: participants confirmed to have early-stage breast cancer; (3) types of studies: randomized controlled trials (RCTs), retrospective studies, and prospective studies were considered; (4) outcome measures: metrics related to surgical procedures, complications, and aesthetic outcomes were included.

The exclusion criteria were as follows: (1) redundantly published articles: studies that appeared in multiple publications were disqualified; (2) studies with incomplete or ambiguous data: manuscripts that presented either incomplete analytical data or inconsistent outcome measures were excluded; (3) substandard quality and unavailability of raw data: research lacking original data or compromised by methodological inadequacies were eliminated; (4) case reports, editorials, expert opinions, and narrative reviews were deemed ineligible for inclusion.

### 2.3 Data extraction

In the meta-analysis, a scrupulous and standardized protocol for data extraction was instituted. Two autonomous reviewers conducted the screening of literature and harvested pertinent data, reciprocally confirming the results for veracity. When discrepancies arose, an internal discussion was engaged amongst the reviewers to reach resolution; consultation with a third expert was invoked if a consensus was unattainable. Datasets extracted encompassed specific variables such as (1) basic characteristics: authorship, year of publication, and nationality; (2) study population: gender, age, sample size, and tumor staging; (3) outcome measures: duration of surgery, intraoperative blood loss, postoperative drainage duration and volume, lymph node yield, and subcutaneous edema. In instances where essential data were not present in the published reports, the primary investigators were solicited via email to obtain any unpublished data fulfilling the study criteria.

### 2.4 Quality assessment

In the meta-analysis, the integrity of the included studies will be stringently appraised by a pair of independent assessors utilizing the Newcastle-Ottawa Scale (NOS) [10]. The NOS serves as a reputable evaluative instrument, stratifying the quality of each study across three pivotal domains: selection criteria, comparability of study groups, and outcome measurement. These domains facilitate a meticulous scrutiny for potential biases intrinsically linked with the included studies. Subsequent to this exhaustive assessment, individual studies are accorded a qualitative score that varies between 0 and 9. The interpretation schema for these scores is delineated thusly: studies that garner a score within the range of 0–3 are classified as low-caliber research, those accumulating a score between 4 and 6 are categorized as moderate-caliber research, and studies that attain a score from 7 to 9 are judged to be high-caliber scholarly work.

### 2.5 Statistical analyses

Inter-study heterogeneity was evaluated through chi-square tests and quantified utilizing the *I* ^2^ statistic. An *I* ^2^ value below 50% accompanied by a corresponding *P*-value equal to or exceeding 0.10 was indicative of non-significant heterogeneity, warranting the use of a fixed-effects model for the amalgamation of effect sizes. Conversely, an *I* ^2^ value of 50% or greater, or a corresponding *P*-value below 0.10, denoted the presence of substantial heterogeneity. Under such circumstances, a random-effects model was employed to estimate the pooled effect size. Sensitivity analyses were conducted to assess the stability of the aggregated results and to identify potential outliers exerting undue influence on the composite effect size. This analytical approach entailed the systematic exclusion of individual studies and subsequent recalculation of the combined effect size. To evaluate the possibility of publication bias, the symmetry of the funnel plot was scrutinized. A symmetrical distribution of data points on both sides of the apex of the funnel plot would imply a lower risk of results being skewed by publication bias. As a quantitative assessment for publication bias, Egger’s linear regression test was administered. All statistical analyses were two-tailed, with a *P*-value below 0.05 considered to signify statistical significance. Data computations were performed using Stata version 17 (StataCorp, College Station, TX, USA).

**Registration:** CRD42024606676.

## 3 Results

### 3.1 Search results and study selection

**Figure 1:**
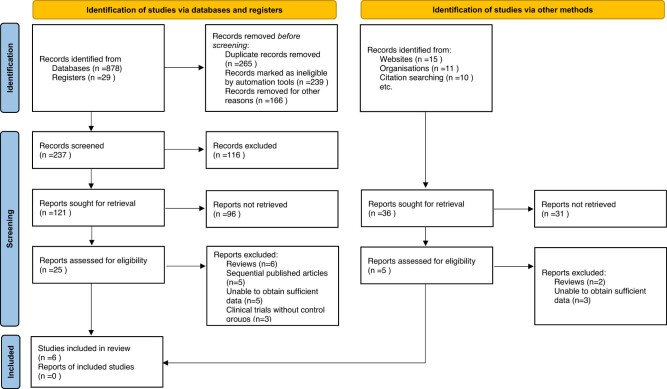
Selection process of included studies.

From the initial search of the electronic databases, 943 related literature were initially found. After removing repetitive literature, reading titles and abstracts, and screening strictly according to the inclusion and exclusion criteria, 30 related literature were obtained, and 24 were excluded from further reading. Finally, six articles were included [7,11–15]. The literature screening process and results are shown in Figure 1.

### 3.2 Study characteristics

The meta-analysis incorporates a total of six studies [7,11–15], spanning from the year 2002 to 2019, and originating from three different countries: China, Italy, and Japan. The studies are predominantly retrospective in nature, with one prospective study and one RCT also included. The sample size for endoscopic interventions ranges from a minimum of 10 to a maximum of 496 patients, while that for conventional treatments varies between 12 and 500 patients. Ages of the participants are either presented as mean ± standard deviation or as a range, and they generally hover around the early to mid-50s for endoscopic treatments and late 40s to early 60s for conventional treatments. Cancer stages represented in the studies are predominantly early-stage, specifically stages I and II, with one study also incorporating stage III (Table 1).

**Table 1:** Summary of included studies in the meta-analysis

First author	Year		Study type	Country	Age mean ± SD or range (endoscopic)	Age mean ± SD or range (conventional)	Number of patients (endoscopic)	Number of patients (conventional)	Cancer stage
Ding et al. [11]	2015		Retrospective	China	47.1 ± 9.1	45.8 ± 11.0	20	20	I, II
Hüscher et al. [12]	2002		Prospective	Italy	56.9 (39–73)	62.7 (38–77)	10	12	I, II
Luo et al. [13]	2012		RCT	China	52.2	47.9	496	500	I, II
Takahashi et al. [7]	2013		Retrospective	Japan	54.2 ± 10.7	61.9 ± 14.3	100	150	I, II, III
Wang et al. [14]	2019		Retrospective	China	51.0 ± 7.9	50.8 ± 7.3	35	35	I, II
Yamashita and Shimizu [15]	2006		Retrospective	Japan	53.7 ± 13.1	50.7 ± 13.0	80	34	I, II

### 3.3 Results of quality assessment

The methodological quality of each incorporated study was rigorously assessed employing the NOS as our evaluation metric. Out of the studies evaluated, one study was allocated a score of 7 points [13], three studies attained 8 points [7,12,15], and two studies achieved the maximum score of 9 points [11,14]. It is noteworthy that none of the studies implemented blinding procedures or exhibited evidence of allocation concealment. Furthermore, there was an absence of any discernible funding biases across all included studies. Additionally, we did not identify any studies with incomplete outcome data, premature termination biases, or imbalances in baseline characteristics. In sum, our quality assessment confirms a high degree of methodological integrity among the majority of the studies included in this meta-analysis, with quality scores ranging between 7 and 9 points. This lends considerable credence to the ensuing analytical processes and interpretative frameworks. Detailed accounts of bias risks and their associated metrics have been compiled and are presented in Table 2.

**Table 2:** Quality assessment according to NOS of each cohort study

Study	Selection				Comparability	Outcome			Total score
	Representativeness of the exposed cohort	Selection of the non-exposed cohort	Ascertainment of exposure	Demonstration that outcome	Comparability of cohorts	Assessment of outcome	Was follow-up long enough	Adequacy of follow-up of cohorts	
Ding et al. [11]	★	★	★	★	★★	★	★	★	9
Hüscher et al. [12]		★	★	★	★★	★	★	★	8
Luo et al. [13]	★	★		★	★	★	★	★	7
Takahashi et al. [7]	★	★	★	★	★★	★		★	8
Wang et al. [14]	★	★	★	★	★★	★	★	★	9
Yamashita and Shimizu [15]	★	★	★	★	★	★	★	★	8

### 3.4 Meta-analysis of operative time between EMIS and traditional surgery for early-stage breast cancer

**Figure 2:**
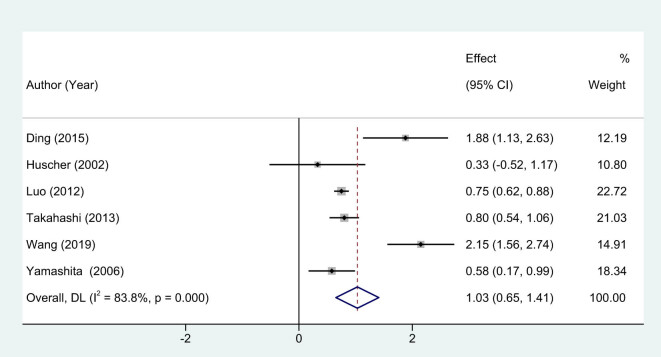
Forest plots of operative time between EMIS and traditional surgery for early-stage breast cancer.

In this meta-analysis, six studies [7,11–15] were included that specifically measured the duration of surgery as one of the key outcome variables. Due to the marked heterogeneity between studies, evidenced by an *I* ^2^ statistic of 83.8%, a random-effects model was deemed appropriate for the calculation of the pooled effect size. The meta-analysis revealed that EMIS is associated with a significantly longer operative time when compared to traditional BCSs. The statistical inference is robust, with a weighted mean difference (WMD) of 1.03 and a 95% confidence interval (CI) ranging between 0.65 and 1.16 (*P* < 0.01, Figure 2).

### 3.5 Meta-analysis of blood loss between EMIS and traditional surgery for early-stage breast cancer

**Figure 3:**
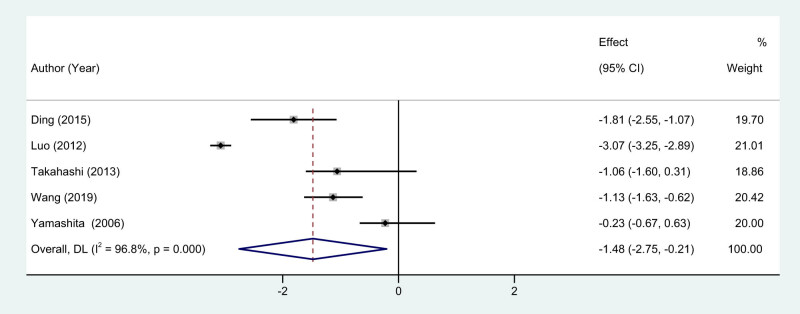
Forest plots of blood loss between EMIS and traditional surgery for early-stage breast cancer.

In the current meta-analysis, a total of five studies [7,11,13–15] were included that reported intraoperative blood loss as a key outcome variable. Given the considerable heterogeneity across these studies, as evidenced by an *I* ^2^ statistic of 96.8%, the implementation of a random-effects model was deemed appropriate for the meta-analytical calculations. The results of the meta-analysis robustly indicate a statistically significant reduction in intraoperative blood loss in the EMIS group when compared to the traditional BCS group. The effect size was represented by a WMD of −1.48, and the 95% CI was in the range of −2.75 to −0.21, substantiating the statistical significance of the findings (*P* < 0.01, Figure 3). Overall, the meta-analysis validates the superiority of EMIS over traditional breast-conserving procedures in terms of reduced intraoperative blood loss, thus underscoring its potential benefits in the clinical setting.

### 3.6 Meta-analysis of drainage volume between EMIS and traditional surgery for early-stage breast cancer

**Figure 4:**
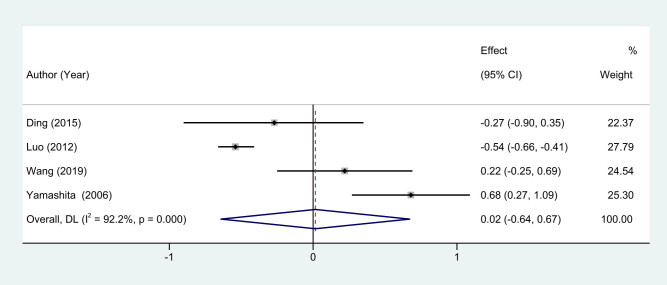
Forest plots of drainage volume between EMIS and traditional surgery for early-stage breast cancer.

In the realm of breast cancer surgeries, the quantity of postoperative drainage serves as an important clinical parameter to evaluate the surgical outcomes and possible complications. To address this, we incorporated drainage volume as one of our primary outcome variables in the current meta-analysis [11,13–15]. The analysis revealed substantial heterogeneity between the included studies, as indicated by an *I* ^2^ statistic of 92.2%. Accordingly, we applied a random-effects model to synthesize the available data for more conservative and robust estimates. Our meta-analysis included four studies that reported on postoperative drainage volume. Notably, the synthesized results did not exhibit a statistically significant difference in drainage volume between the EMIS group and the traditional BCS group. The overall WMD was 0.02, with a 95% CI spanning from −0.64 to 0.67, and a *P*-value exceeding 0.05 (Figure 4). These findings suggest that both surgical techniques appear to be comparable in terms of postoperative drainage volume, thereby offering no distinct advantage or disadvantage for either procedure in this specific aspect.

### 3.7 Meta-analysis of lymph node dissection counts between EMIS and traditional surgery for early-stage breast cancer

**Figure 5:**
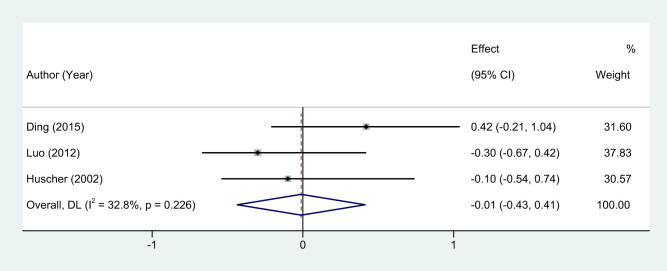
Forest plots of lymph node dissection counts between EMIS and traditional surgery for early-stage breast cancer.

The efficacy of lymph node harvest during surgical intervention for breast cancer is a paramount indicator for assessing the quality of oncological surgery. In this meta-analysis, we evaluated whether EMIS offers any benefit over traditional surgical procedures in terms of lymph node harvest. Three studies [11–13] were identified that specifically included lymph node harvest as an outcome metric. Unlike other evaluated parameters, the studies on this metric exhibited low heterogeneity, as indicated by an *I* ^2^ statistic of 32.8%. This allowed us to employ a fixed-effects model for the quantitative synthesis of effect sizes. Upon analysis, the meta-analysis revealed no statistically significant difference in the number of lymph nodes harvested during the procedure between the EMIS and traditional surgery groups. The calculated WMD was −0.01, falling within a 95% CI range of −0.43 to 0.41. The resultant *P*-value was greater than 0.05, reinforcing the lack of statistical significance (Figure 5). The findings underscore that both EMIS and traditional surgical methods appear to be equivalently effective in harvesting lymph nodes during surgical intervention.

### 3.8 Sensitivity analysis to evaluate the robustness of pooled outcomes in the meta-analysis

**Figure 6:**
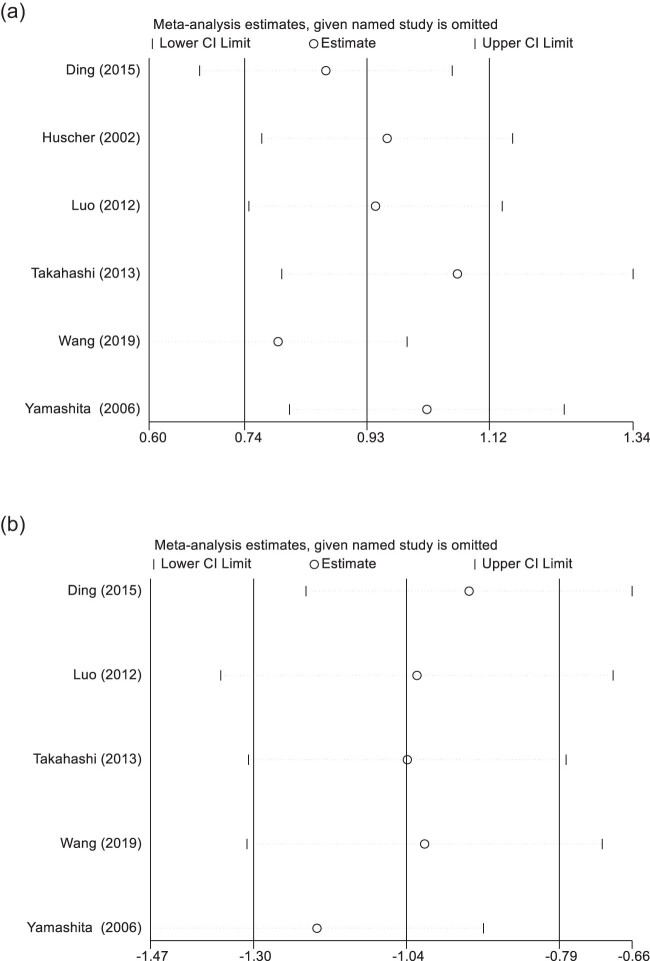
Sensitivity analysis of the operative time (a) and blood loss (b) between EMIS and traditional surgery for early-stage breast cancer.

In light of the significant heterogeneity encountered among the incorporated studies, we executed a sensitivity analysis to rigorously evaluate the robustness and dependability of the aggregated effect sizes [7,11–15]. The procedure entailed a serial omission of each study from the meta-analytic model, followed by a recalculation of the combined effect measures using the residual studies. This meticulous sensitivity assessment substantiated that the cumulative outcomes were not unduly impacted by any single study’s exclusion, confirming their stability and reliability. Such an observation corroborates that no individual study wielded a disproportionate influence on the pooled results, thereby bolstering the credibility of our meta-analytic conclusions. The steadfastness of outcomes across these sensitivity tests accentuates the resilience of our principal findings, further reinforcing the inferences made in this meta-analysis (Figure 6).

### 3.9 Assessment of publication bias through funnel plot and Egger’s test

**Figure 7:**
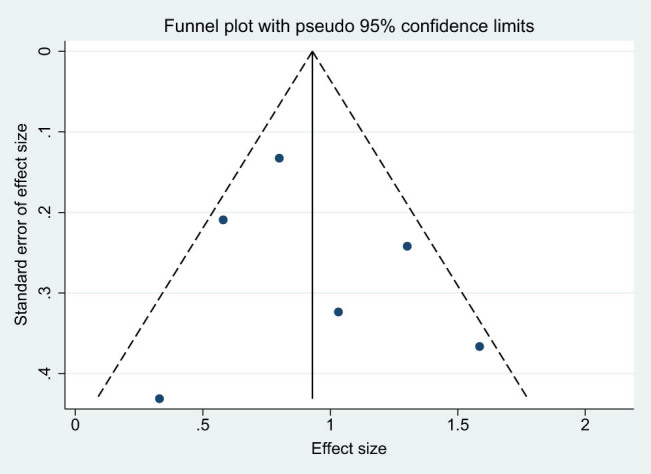
Funnel plot for publication bias.

The construction of funnel plots using the studies [7,11–15] incorporated into the meta-analysis exhibited symmetrical distributions, providing no evidence of substantial publication bias (Figure 7). Additionally, Egger’s linear regression analysis corroborated the absence of any statistically significant publication bias across the evaluated variables (*P*-value exceeding 0.05 for each variable assessed). This lends further credence to the stability and validity of the results generated by this meta-analysis.

## 4 Discussion

This meta-analysis introduces significant contributions to the existing body of evidence comparing EMIS and traditional surgery in the management of early-stage breast cancer. The novelty of this study lies in its comprehensive aggregation and analysis of comparative data across multiple countries and study designs, which enhances the generalizability of the findings. Clinically, our results underscore the superiority of EMIS in reducing intraoperative blood loss, a crucial factor in enhancing patient recovery and reducing hospital stay durations. Furthermore, the comparable outcomes between EMIS and traditional surgery regarding operative time, postoperative drainage, and lymph node dissection underline the versatility and safety of EMIS. This evidence supports the potential for EMIS to be integrated as a standard care option, offering a less invasive alternative without compromising therapeutic efficacy. The study’s implications extend to surgical practice, suggesting that healthcare systems should consider broader adoption and facilitation of EMIS, which could lead to significant improvements in patient-centered outcomes and resource utilization in breast cancer care.

Breast cancer has emerged as one of the most widespread malignancies affecting women worldwide. Most of these cases, especially in the beginning, can achieve clinical remission by undergoing surgery together with additional treatment with radiation and chemotherapy [16,17]. The rate of DFS after surgery for early-stage breast cancer is relatively high. The importance of the breast in preserving a woman’s body image is extremely significant, which supports the growing use of breast-conserving operations. Aside from its aesthetic advantages, BCS significantly enhances the quality of life in patients after tumor removal. Endoscopic procedures in breast surgery are increasingly becoming used in clinical practice. These methods not only achieve the best possible cancer treatment results, but they also provide better aesthetic results after surgery [18,19].

The findings of our meta-analysis suggest that there is no statistically significant disparity in postoperative drainage volume and lymph node dissection counts between EMIS and standard surgical techniques. This indicates that endoscopic methods do not cause an increase in the amount or duration of drainage after surgery. The improved visibility offered by the endoscopic method allows for more effective removal of lymph nodes and control of bleeding. Moreover, this work showcases that endoscopic procedures have the ability to substantially decrease intraoperative blood loss. The minimally invasive aspect of endoscopic BCS enables surgical intervention through smaller incisions, which is the reason for this outcome. By employing this technique, the harm inflicted upon the skin, muscle, and other bodily tissues is minimized, leading to a decrease in the amount of blood lost in comparison to conventional procedures. Furthermore, it is crucial to acknowledge that endoscopic breast surgery necessitates a heightened level of surgical skill. The duration of the procedure can be significantly extended as compared to conventional surgical techniques, necessitating intensive training and practical experience for physicians to acquire proficiency in these particular abilities.

The breast, as a prominent secondary sexual feature in women, possesses psychological importance that extends beyond its physiological roles. During breast cancer treatment, individuals have psychological requirements related to the aesthetics of their breasts, such as reducing surgical scars and maintaining the integrity of the nipple-areola complex. Endoscopic surgery can significantly fulfill these aesthetic objectives when compared to conventional surgical approaches. Conventional BCS, while maintaining the breast, frequently results in noticeable scarring on the surface of the breast. Surgical incisions can have a maximum length of 10 cm, and it is common to require further incisions in the axillary region [20]. These aspects are incongruent with the patient’s aesthetic preferences and expectations. Patients experience both physical pain resulting from surgical trauma and psychological suffering produced by postoperative scarring. The latter can have significant consequences, impacting not just the individual’s self-perception but also their sexual and marital relationships. The endoscopic technique provides a benefit in this aspect by reducing the visibility of surgical scars, thereby more closely meeting the cosmetic expectations of patients. By deliberately placing smaller incisions, this surgical approach minimizes postoperative scarring, hence reducing both physical and psychological trauma to the patient. Furthermore, the endoscopic procedure enables superior preservation of the nipple-areola complex, a crucial element that contributes to the overall aesthetic and psychological well-being of the patient. Preserving the nipple-areola complex is particularly crucial in early-stage breast cancer cases where the tumor is not close to it and can be feasibly saved [21,22].

The presence of heterogeneity in meta-analyses is a multifaceted problem that arises from various sources. This makes it challenging to combine the data of different studies and might potentially impact the reliability of the conclusions drawn. Methodological heterogeneity occurs due to differences in study designs, such as the use of RCTs vs cohort studies, which impact the internal validity of the overall conclusions. This is further complicated by the presence of clinical heterogeneity, which is caused by differences in patient characteristics, disease severity, and the presence of other medical disorders. Additionally, there are differences in the locations where treatments are given, such as in hospitals or outpatient clinics. The presence of geographical and temporal heterogeneity contributes to the variety observed in research outcomes. This variability is a result of disparities in healthcare infrastructure, medical practices, and cultural perspectives on treatment that are influenced by place and time period. Outcome measure heterogeneity arises when research utilizes a variety of metrics or scales to assess results, making it difficult to effectively combine and analyze the data. All of these characteristics has a role in the total complexity and should be thoroughly taken into account when conducting and analyzing meta-analyses.

It ought to recognize certain constraints when evaluating the findings of this meta-analysis. Initially, the criteria for inclusion may have limited the variety of study designs and methodologies, which could have led to selection bias and reduced the applicability of the results. Furthermore, the presence of substantial variation among the research considered may undermine the accuracy of combined estimates, despite the implementation of sensitivity analysis. Furthermore, it is possible that there is publication bias that has not been taken into consideration, even when funnel plots and statistical tests have been employed. This bias may arise because research that have negative or null results are less inclined to be published. Furthermore, this study was constrained by the fact that we only had access to publically available information for data extraction, and we were unable to obtain specific patient data. As a result, the level of detail in our analysis was limited. Furthermore, discrepancies in healthcare environments and procedures among various geographical regions can complicate the results, posing challenges in the generalizability of the findings. These constraints require a careful study of the meta-analysis and emphasize areas that need further investigation.

## 5 Conclusions

In conclusion, our systematic review and meta-analysis demonstrate that EMIS for the treatment of early-stage breast cancer offers distinct advantages over traditional breast-conserving procedures. While the operative time for the endoscopic approach may be longer, it is offset by a reduction in intraoperative blood loss. Furthermore, our data reveal no statistically significant differences in postoperative drainage volume or the number of lymph nodes dissected when compared to traditional surgical methods. Importantly, endoscopic minimally invasive techniques yield more aesthetically pleasing results for the breast, thus fulfilling a crucial aspect of patient-centered care.

## Appendix

**Table A1:** Search strategies utilised for the database search

Database	Search Strategies
PubMed	("early-stage breast cancer" OR "stage I breast cancer" OR "stage II breast cancer") AND ("endoscopic minimally invasive surgery" OR "minimally invasive surgery" OR "laparoscopy") AND ("traditional surgery" OR "modified radical mastectomy" OR "breast-conserving surgery")
	**Filters applied:** No language restrictions; study types included randomized controlled trials (RCTs), retrospective studies (RS), and prospective studies (PS)
Embase	("early-stage breast cancer" OR "stage I breast cancer" OR "stage II breast cancer") AND ("endoscopic minimally invasive surgery" OR "minimally invasive surgery" OR "laparoscopy") AND ("traditional surgery" OR "modified radical mastectomy" OR "breast-conserving surgery")
	**Filters applied:** No temporal restrictions; studies published in English and other languages were included. Only human studies and RCTs, prospective, and retrospective studies were considered
Web of Science	("early-stage breast cancer" OR "stage I breast cancer" OR "stage II breast cancer") AND ("endoscopic minimally invasive surgery" OR "laparoscopy" OR "minimally invasive surgery") AND ("traditional surgery" OR "modified radical mastectomy" OR "breast-conserving surgery")
	**Filters applied:** English language; no time restrictions; only peer-reviewed articles and clinical studies
Cochrane Library	("early-stage breast cancer" OR "stage I breast cancer" OR "stage II breast cancer") AND ("endoscopic minimally invasive surgery" OR "laparoscopy" OR "minimally invasive surgery") AND ("traditional surgery" OR "modified radical mastectomy" OR "breast-conserving surgery")
	**Filters applied:** No language restrictions; included systematic reviews, clinical trials, and comparative studies
