# Corrigendum to “Comparing the therapeutic efficacy of endoscopic minimally invasive surgery and traditional surgery for early-stage breast cancer: A meta-analysis”

**DOI:** 10.1515/med-2025-9994

**Published:** 2025-04-17

**Authors:** Qiji Ma, Tingting Shi, Huan Wang, Jie Xing

**Affiliations:** Department of Breast and Nail Surgery, The Affiliated Hospital to Changchun University of Chinese Medicine, Changchun, 130021, Jilin, China; Treatment Center, The Affiliated Hospital to Changchun University of Chinese Medicine, Changchun, 130021, Jilin, China; Rehabilitation Department, The Affiliated Hospital to Changchun University of Chinese Medicine, Changchun, 130021, Jilin, China; Department of Breast and Nail Surgery, The Affiliated Hospital to Changchun University of Chinese Medicine, 1478 Gongnong Road, Changchun, 130021, Jilin, China

In the published article Ma Q, Shi T, Wang H, Xing J. Comparing the therapeutic efficacy of endoscopic minimally invasive surgery and traditional surgery for early-stage breast cancer: A meta-analysis. Open Med. (Wars) 2025; 20(1):20241133, doi: 10.1515/med-2024-1133; PMID: 39844785; PMCID: PMC11751671, there is an error in the first author surname: Qiyi Ma. The correct author name and surname is as follows: Qiji Ma.

